# Comparison of Super Resolution Reconstruction Acquisition Geometries for Use in Mouse Phenotyping

**DOI:** 10.1155/2013/820874

**Published:** 2013-09-23

**Authors:** Niranchana Manivannan, Bradley D. Clymer, Anna Bratasz, Kimerly A. Powell

**Affiliations:** ^1^Department of Electrical and Computer Engineering, The Ohio State University, Columbus, OH 43210, USA; ^2^Small Animal Imaging Shared Resources, The Ohio State University, Columbus, OH 43210, USA; ^3^Department of Biomedical Informatics, The Ohio State University, Columbus, OH 43210, USA

## Abstract

3D isotropic imaging at high spatial resolution (30–100 microns) is important for comparing mouse phenotypes. 3D imaging at high spatial resolutions is limited by long acquisition times and is not possible in many *in vivo* settings. Super resolution reconstruction (SRR) is a postprocessing technique that has been proposed to improve spatial resolution in the slice-select direction using multiple 2D multislice acquisitions. Any 2D multislice acquisition can be used for SRR. In this study, the effects of using three different low-resolution acquisition geometries (orthogonal, rotational, and shifted) on SRR images were evaluated and compared to a known standard. Iterative back projection was used for the reconstruction of all three acquisition geometries. The results of the study indicate that super resolution reconstructed images based on orthogonally acquired low-resolution images resulted in reconstructed images with higher SNR and CNR in less acquisition time than those based on rotational and shifted acquisition geometries. However, interpolation artifacts were observed in SRR images based on orthogonal acquisition geometry, particularly when the slice thickness was greater than six times the inplane voxel size. Reconstructions based on rotational geometry appeared smoother than those based on orthogonal geometry, but they required two times longer to acquire than the orthogonal LR images.

## 1. Introduction

MRI is being used more frequently for evaluating morphological phenotypes in genetically engineered mouse models of disease [1]. 3D imaging at the highest spatial resolution is the preferred approach for comparing morphological phenotypes; however, it is not always possible in small animal *in vivo* imaging settings. This is due to the long acquisition times required to achieve high spatial resolution. Several-factors limit obtaining high-resolution 3D isotropic images in the *in vivo* settings such as the length of time a mouse can be kept under anesthesia, motion artifacts that are likely to occur during long acquisition protocols that degrade image quality, and increased repetition times required at the high magnetic field strengths used for small animal imaging. Keeping animals under anesthesia for long periods of time (>2 hrs) is not desirable. MRI acquisition protocols with very long repetition times (*T*_*R*_ > 1500 ms), such as T2-weighted, diffusion-weighted (DW), and inversion recovery imaging are particularly affected by the long scan times required for 3D isotropic imaging. Thus, *in vivo* MR images in small animal studies are usually acquired using 2D multislice acquisitions with inplane resolutions (50–100 *μ*m) which are 5–10 times greater than the resolution in the slice-select direction (500–1000 *μ*m).

2D multislice images suffer from the effects of partial volume averaging due to their increased slice thickness, and when reformatted and viewed from a perspective other than the inplane acquisition direction, the features often appear blurry due to decreased resolution in the slice-select direction. Increasing the resolution in the slice-select direction comes at the expense of decreased signal-to-noise ratio (SNR) due to the smaller voxel size. Signal-to-noise ratio is directly proportional to voxel size and the square root of number of signal averages. Therefore, in order to compensate for a decrease in SNR due to a decrease in voxel size, the number of signal averages must be increased by a factor proportional to the decrease in voxel size and thus a proportional increase in acquisition time. Decreasing the slice thickness also requires increasing the number of slices in order to cover the same FOV which also results in increased acquisition time. This trade-off between spatial resolution, acceptable SNR, and image acquisition time is always a consideration when imaging live subjects. MRI acquisition techniques, such as parallel imaging [2] and partial Fourier imaging [3] have been proposed for speeding up acquisition times so that higher resolution images can be acquired. These techniques require specialized hardware and software for implementation and are not always available for small animal MRI applications. Super resolution reconstruction (SRR) is an image postprocessing approach that has been proposed to improve the resolution in the slice-select direction in 2D multislice MRI data set [4]. It is based on reconstructing a high-resolution (HR) image from a set of low-resolution (LR) image stacks that were obtained from different viewpoints of the same field-of-view (FOV). Its application is not limited by the availability of acquisition hardware or software and can be used in any multislice acquisition setting including those that utilize high-speed acquisition protocols, such as parallel or partial Fourier imaging. 

The SRR approaches proposed this far for MRI have differed primarily in the orientation of the acquisition geometry of the set of LR image stacks and the iterative optimization technique used for SRR. Greenspan et al. [4] proposed collecting a set of LR image stacks by subpixel shifting the 2D multislice stack acquisitions in the slice-select direction. Irani and Peleg's iterative backprojection method (IBP) [5] was then used to reconstruct the HR image from the shifted LR stacks. For this method, the number of LR image stacks required to reconstruct an isotropic 3D HR image is directly related to the ratio of the slice thickness to the inplane resolution of the LR images. Thus, the more anisotropic the LR data acquisitions are the greater the number of LR image stacks are required. Shilling et al. [6] proposed acquiring a set of LR image stacks by rotating the slice-select direction in equal angle sampling intervals about a central axis. Six LR image stacks, obtained at 30° rotational increments, were used for SRR. Additive and multiplicative algebraic reconstructions were used to reconstruct the HR image from the LR image stacks. Additive correction was found to be better than the multiplicative method for high noise levels. Resolution enhancement was observed in phantom studies, *ex vivo*, and human brain scans. Souza and Senn [7] based their SR reconstructions on the acquisition of three orthogonal (i.e., coronal, sagittal, and axial) LR image stacks. IBP was also used for reconstructing HR images from the LR image stacks in this approach. Qualitative and quantitative evaluations indicated that SRR using LR image stacks acquired orthogonally might be useful for improving spatial resolution and contrast-to-noise ratios (CNR) similar to that observed using shifted and rotational geometries. Recently, Plenge et al. [8] evaluated the different optimization techniques used for SRR of MRI data. Their results indicated that reconstruction methods based on IBP and least squares optimization techniques performed better than those based on algebraic reconstruction. Plenge's evaluation was performed using the rotational acquisition geometry proposed by Shilling et al. [6]. No evaluation of the effect of LR acquisition geometry on SRR has been performed. 

Our overall goal was to determine whether SRR with a minimal number of LR views would be useful for morphological evaluations of *in vivo* animal models. In order for SRR to be applicable in small animal phenotyping applications, the LR image stacks must be acquired in significantly less time than a comparable HR 3D isotropic acquisition, and the SRR image should have comparable image quality to that observed in images obtained from a HR acquisition. To achieve this goal, we investigated the effect LR acquisition geometry (shifted, rotation, and orthogonal) and the number of LR image stacks with different voxel aspect ratios (AR) have on SRR. A voxel's AR refers to the proportional relationship of its size in each dimension (i.e., width : height : depth) and is directly related to SNR and acquisition time. For this study, quantitative and qualitative evaluations of SRR images were performed using a resolution (line pair) and a biological (*ex vivo* embryo) phantom. Image quality was assessed by comparing the SRR images to a HR 3D isotropically acquired image. SRR was also implemented for an *in vivo* animal imaging application.

## 2. Materials and Methods

### 2.1. Super Resolution Reconstruction Method

**Figure 1:**
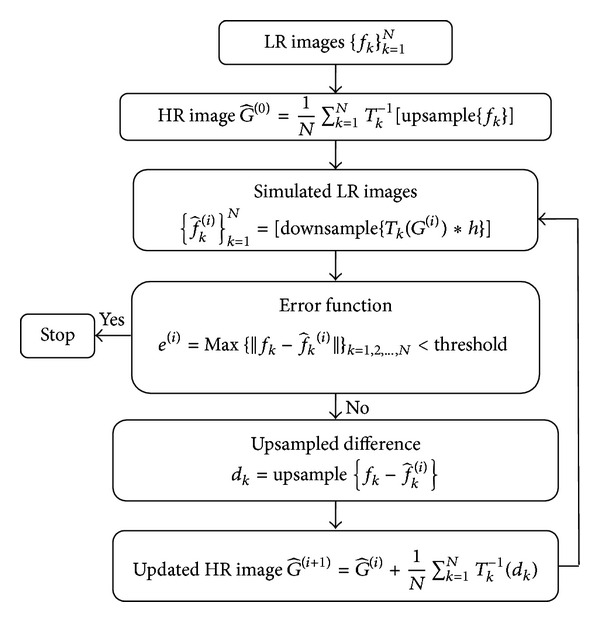
Block diagram of Irani and Peleg's IBP algorithm.

All SRR images were reconstructed using the IBP approach proposed by Irani and Peleg [5]. IBP was chosen because it has been widely used for super resolution reconstruction in the past and because of its easy implementation. A flowchart illustrating the IBP approach is provided in Figure 1. Initially, an HR image ${\hat{G}}^{\left(0\right)}$ is approximated from the average of multiple LR images {*f*_*k*_}_*k*=1_^*N*^ that have been geometrically transformed, *T*_*k*_^−1^, to the same orientation prior to averaging. A new set of LR images ${\left\{{{\hat{f}}_{k}}^{\left(0\right)}\right\}}_{k=1}^{N}$, are obtained by simulating the imaging process (blurring *h*, and down sampling) in the predicted HR image ${\hat{G}}^{\left(0\right)}$. For our case, a 1D Gaussian kernel with a FWHM equal to the LR slice thickness was used along the slice-select direction in the HR image for blurring because it closely matched the excitation profile used in the original image acquisition sequence. If the predicted HR image ${\hat{G}}^{\left(0\right)}$ is the same as the true HR image *G*, then the simulated LR images ${\left\{{{\hat{f}}_{k}}^{\left(0\right)}\right\}}_{k=1}^{N}$ should be equal to the observed LR images {*f*_*k*_}_*k*=1_^*N*^. Therefore, the difference between the observed and simulated LR images ${\left\{f_{k}-{{\hat{f}}_{k}}^{\left(0\right)}\right\}}_{k=1}^{N}$ is upsampled and backprojected on to ${\hat{G}}^{\left(0\right)}$ using linear interpolation. This results in an updated HR image ${\hat{G}}^{\left(1\right)}={\hat{G}}^{\left(0\right)}+(1/k)\sum_{k=1}^{N}\left(\text{upsample}\left\{f_{k}-{{\hat{f}}_{k}}^{\left(0\right)}\right\}\right)$ that can be downsampled and the simulated LR images ${\left\{{{\hat{f}}_{k}}^{\left(i\right)}\right\}}_{k=1}^{N}$ are compared to the observed LR images {*f*_*k*_}_*k*=1_^*N*^. These steps are iteratively repeated till the maximum error at the *i*th iteration according to $e^{\left(i\right)}={\mathrm{Max}\left\{||f_{k}-{{\hat{f}}_{k}}^{\left(i\right)}||\right\}}_{k=1,2,..,N}$ is less than a preset threshold. All SRR software was developed using Matlab v.2009a (MathWorks Inc., Mass, USA).

### 2.2. MR Image Acquisition

#### 2.2.1. Resolution Phantom

**Figure 2:**
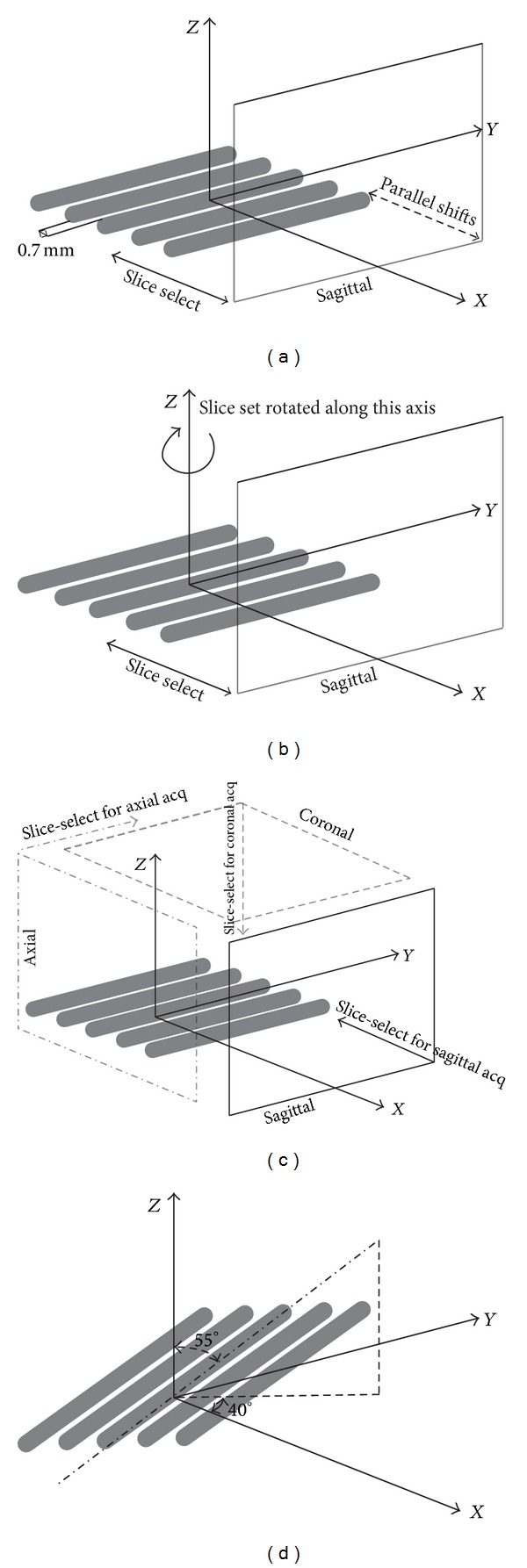
Schematic illustrating the orientation of the resolution phantom where the long axis of tubes were positioned orthogonally to the slice-select direction of the (a) shifted, (b) rotational, (c) orthogonal acquisition geometries, and (d) orientation of tubes in the resolution phantom for the oblique setup and the acquisition geometries shown in Figures 2(a), 2(b), and 2(c) were repeated for this oblique orientation. (Axes in this image represent physical coordinates and the main magnetic field is in Z direction).

A resolution phantom was constructed using five cylindrical quartz EPR tubes (0.5 mm ID, 0.7 mm OD). The tubes were cut into 2.5 cm lengths and were placed side-by-side with a known separation of 0.7 mm (see illustration in Figure 2(a)). The tubes were sealed with the air trapped inside them, resulting in a signal void within the tubes. They were then immersed in the center of a 15 mL tube (14 mm ID) filled with 1 : 30 (v : v) homogeneous mixture of gadopentetate dimeglumine (GD) Magnevist (Bayer Pharmaceutical, Wayne NJ) and water.

**Figure 3:**
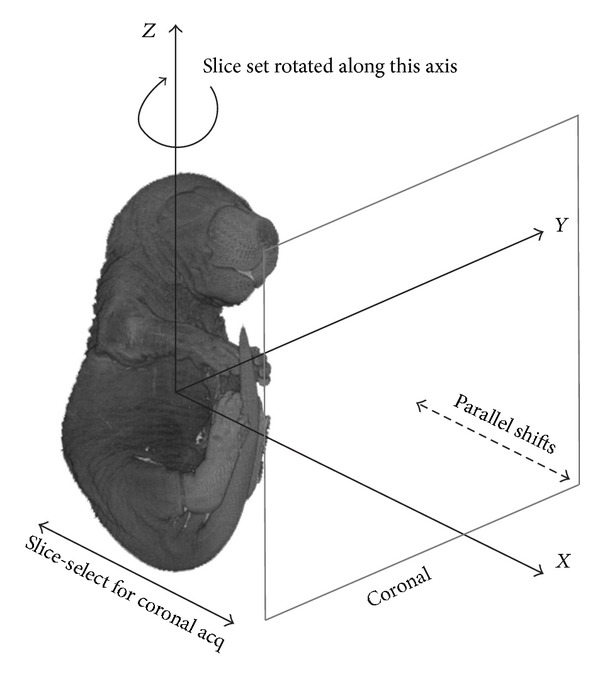
Schematic illustrating the orientation of the *ex vivo *embryo with respect to the slice-select direction of the acquisition geometries.

LR image stacks of the phantom were acquired using a Bruker Biospin Avance 500 MHz 11.7T magnet (Bruker Biospin, Karlsruhe, Germany) and a 25 mm diameter volume coil and a T1-weighted FLASH imaging sequence (*T*_*R*_ = 348.2 ms, *T*_*E*_ = 6 ms, FA = 90°, FOV = 2.6 × 2.6 cm, 1 mm slice thickness, navgs = 4, number of contiguous slices = 26, and acquisition time = 4 m 56 s). The phantom was imaged at two orientations relative to the slice-select direction of the three acquisition geometries. The first orientation was where the long axis of the tubes were positioned along the *Y*-axis as illustrated in Figures 2(a)–2(c). The second orientation was where the long axis of the tubes were positioned obliquely to the slice-select direction of the acquisition geometries as illustrated in Figure 2(d). For this orientation the tubes were rotated 40° in the *XY*-plane and 55° in the *YZ*-plane in the oblique orientation. The oblique orientation represents the most extreme case where edge reconstruction is affected due to partial volume averaging in the slice-select direction. LR image stacks were collected using an inplane matrix size of 128 × 128 and 256 × 256 for voxel ARs of 1 : 1 : 5 and 1 : 1: 10, respectively. For both orientations mentioned above, the stacks were obtained using the following three acquisition geometries: (1) five sets of LR image stacks were acquired using 0.20 mm subpixel shifts in the slice-select direction for voxel AR of 1 : 1 : 5 and ten sets were acquired using 0.10 mm subpixel shifts in the slice-select direction for voxel AR of 1 : 1: 10 (shifted) (Figure 2(a)), (2) six sets were acquired with 30° angular rotations along the slice-select direction for both ARs of 1 : 1 : 5 and 1 : 1: 10 (rotated) (Figure 2(b)), and (3) three sets were acquired orthogonally to one another in axial, coronal, and sagittal planes for both ARs of 1 : 1 : 5 and 1 : 1: 10 (orthogonal) (Figure 2(c)). SRR images were calculated for each acquisition geometry using the SRR method described above. 

The quality of the SRR was evaluated by visual inspection of the resolution phantom in the short axis view (i.e, short axis of the tubes) where blurring in the slice-select direction is expected to be the greatest due to the low resolution sampling in that direction. Intensity line plots were obtained to better visualize the effects of SRR on signal intensity and edge transitions. SRR images were compared to a high resolution image of the phantom acquired in the axial plane. 

#### 2.2.2. Biological Phantom

An *ex vivo* E17.5 wild type embryo was used as a biological phantom for evaluating the effects of SRR on live subject MRIs. It possesses anatomic structures similar to that observed in live animals and does not suffer from motion artifacts observed in *in vivo* imaging. It is also possible to obtain an isotropic high resolution volume image of the *ex vivo* embryo for comparison to the SRR images. The E17.5 embryo was fixed and stained for 2 hours using a 20 : 1 volume ratio of 4% paraformaldehyde and PBS : GD solution. It was then stabilized and stored in 15 mL of 200 : 1 PBS : GD solution prior to imaging. For MR imaging, the embryo was suspended in a 15 mL tube of Fluorinert FC-70(3M Company, St. Paul MN). 

 The LR image stacks of the *ex vivo* embryo were obtained using a Bruker Biospin Avance 500 MHz 11.7T magnet (Bruker Biospin, Karlsruhe, Germany) and a 25 mm diameter volume coil and T1-weighted FLASH imaging sequence (*T*_*R*_ = 519.5 ms, *T*_*E*_ = 4 ms, FA = 30.0, FOV = 2.2 × 2.2 cm, matrix = 512 × 512, navgs = 1, and acquisition time = 3 min) and two different slice thicknesses, 0.19 mm (voxel AR = 1 : 1 : 4, number of contiguous slices = 64) and 0.26 mm (voxel AR = 1 : 1 : 6, number of contiguous slices = 46). Two additional slice thicknesses were evaluated for the orthogonal acquisition geometry, 0.38 mm (voxel AR = 1 : 1 : 8, number of contiguous slices = 32) and 0.46 mm (voxel AR = 1 : 1 : 10, number of continguous slices = 26). LR image stacks were obtained using the acquisition geometries outlined above: (1) four sets of LR image stacks were acquired using 0.0475 mm subpixel shifts in the slice-select direction for a voxel AR 1 : 1 : 4 and six sets were acquired using 0.0433 mm subpixel shifts in the slice-select direction for a voxel AR 1 : 1 : 6 (shifted), (2) six sets were acquired with 30° angular rotations along the slice-select direction for both ARs of 1 : 1 : 4 and 1 : 1 : 6 (rotated), and (3) three sets were acquired orthogonal to another in axial, coronal, and sagittal planes for ARs of 1 : 1 : 4, 1 : 1 : 6, 1 : 1 : 8, and 1 : 1: 10 (orthogonal). The embryo was positioned such that the subpixel shifts were done along the *X* axis for the shifted geometry and angular rotations were done around the *Z* axis for the rotational geometry (Figure 3). SRR images were calculated for each LR acquisition geometry using the SRR method described above.

 3D isotropic volume images of the same embryo were acquired for comparison to the SRR images. A T1-weighted 3D FLASH sequence (*T*_*R*_ = 11.3 ms, *T*_*E*_ = 4.0 ms, FA = 20.0, FOV = 2.2 × 2.2 × 1.2 cm, matrix = 512 × 512 × 256, navgs = 1, acqusition time = 18.5 min) was used for the 3D imaging. The 3D image obtained from this acquisition protocol results in a high-quality image that is routinely used for biological phenotyping of *ex vivo* embryos in our laboratory.

#### 2.2.3. Quantitative Measures

**Figure 4:**
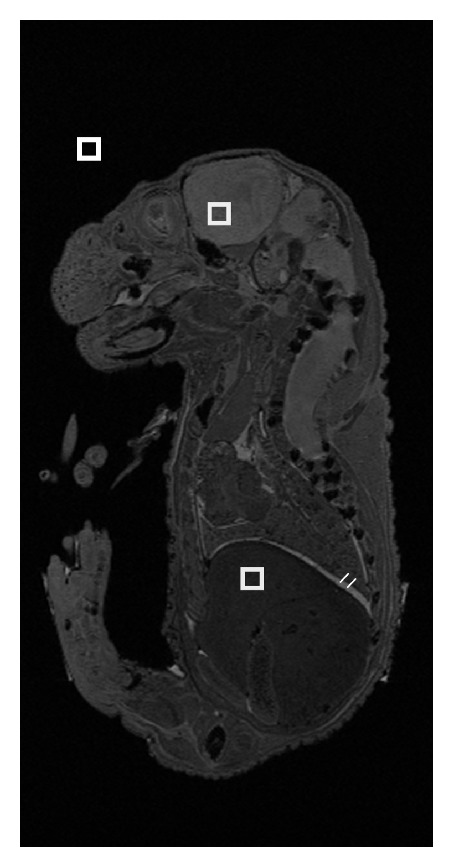
2D slice image of the *ex vivo *embryo illustrating the location of 9 × 9 × 9 voxel ROI chosen for SNR and CNR calculations and sample edge profiles chosen to calculate the edge width.

SRR images were qualitatively compared to the isotropically acquired 3D image of the biological phantom. SNR, contrast-to-noise ratio (CNR), and edge pixel width were used for quantitative evaluation of the SRR images. SNR and CNR were calculated using 9 × 9 × 9 voxel regions within homogenous regions of tissue illustrated in Figure 4. SNR was calculated using the following equation:

(1) $$\begin{array}{c} \text{SNR}=\frac{S}{\sigma_{n}}, \end{array}$$

where *S* = mean signal intensity (regions selected in brain as shown in Figure 4) and *σ*_*n*_ = standard deviation of the noise (from background as shown in Figure 4). CNR was calculated using the following equation:

(2) $$\begin{array}{c} {\text{CNR}}_{hl}=\frac{\left|S_{h}-S_{l}\right|}{\mathrm{Max}\left(\sigma_{h},\sigma_{l}\right)}, \end{array}$$

where *S*_*l*_, *S*_*h*_ and *σ*_*l*_, *σ*_*h*_ are mean signal intensity and standard deviation in low and high signal intensity ROIs.

Edge profiles were measured by nonlinear least-square fitting of a sigmoid function of the form [4, 6, 8]

(3) $$\begin{array}{c} f=a_{1}+\frac{a_{2}}{1+\exp\left(-a_{3}\left(x-a_{4}\right)\right)}. \end{array}$$

The edge width in high resolution pixels is computed by

(4) $$\begin{array}{c} \text{Edge Width }\left[\text{Pixels}\right]=\frac{4.4}{a_{3}}. \end{array}$$

This corresponds to the rise length from 0.1 to 0.9 of the normalized values, when *a*_3_ = 4.4, it corresponds to the rise length of one voxel. An estimate of resolution can be obtained from these edge widths. The mean edge width was calculated from 20 edge profiles obtained across the liver boundary as illustrated in Figure 4.

#### 2.2.4. *In Vivo* Mouse

MR imaging of a live mouse was performed using a Bruker Biospin Avance 400 MHz 9.4T magnet (Bruker Biospin, Karlsruhe, Germany). All animal protocols were approved by the Institutional Laboratory Care and Use Committee of the Ohio State University. The mouse was placed prone on a temperature controlled mouse bed and inserted into the 35 mm diameter quadrature volume coil. The mouse was anesthetized with 2.5% isoflurane mixed with 1 liter per minute carbogen and maintained with 1–1.5% isoflurane during imaging. The respiration and temperature of the animal were monitored during the course of the experiment using a Small Animal Monitoring and Gating System (Model 1025, Small Animals Instruments, Inc. Stony Brook, NY). A bolus of 11 *μ*L of 11.2 mg iron oxide I.V. (Feridex, AMAH Pharmaceuticals, Lexington MA) per 1 mL PBS was injected via tail vein approximately 20 min prior to imaging. An orthogonal set of LR image stacks (voxel AR of 1 : 1 : 10) of the live mouse was acquired using a respiratory-gated T1-weighted FLASH imaging sequence (TR = 200 ms, TE = 2.72 ms, FA = 55.0, FOV = 2.5 cm × 2.5 cm, navgs = 8, FOV = 2.5 × 2.5, matrix = 256 × 256, 1 mm slice thickness, acqusition time = 15 min). Contiguous slices covering 25 mm of the upper abdominal region were acquired. 

## 3. Results

### 3.1. Resolution Phantom

**Figure 5:**
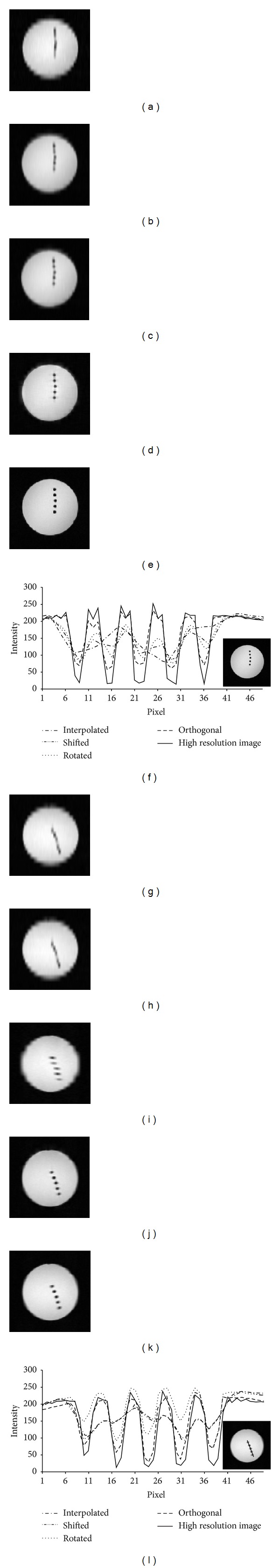
2D slice images (image plane represented is orthogonal to the long axis of the tube which is placed along *Y*-axis in Figure 2(a)) of resolution phantom where the long axis of the tubes is orthogonal to the acquisition plane, and LR image stacks were collected with a voxel AR of 1 : 1 : 5: (a) interpolated, (b) shifted, (c) rotational, (d) orthogonal, (e) inplane, and (f) line plot, and where the long axis of the tubes is oblique to the acquisition plane: (g) interpolated, (h) shifted, (i) rotational, (j) orthogonal, (k) inplane, and (l) line plot.

**Figure 6:**
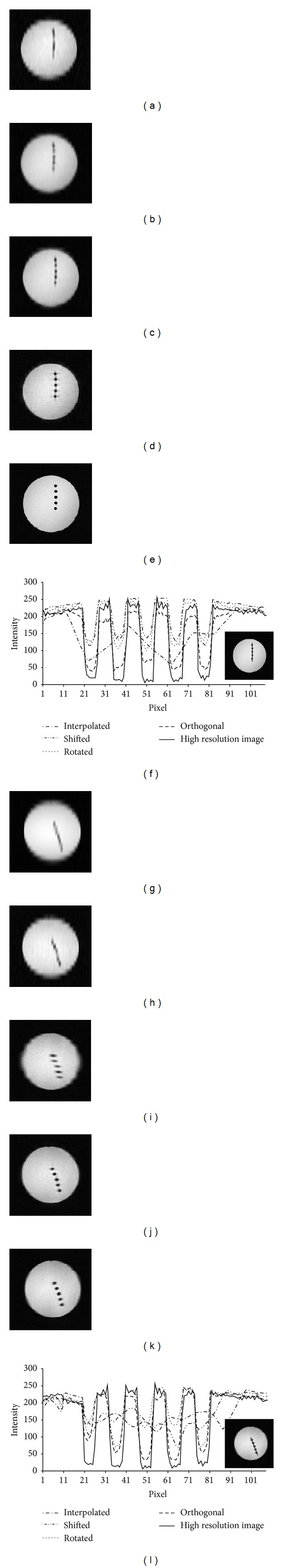
2D slice images (image plane represented is orthogonal to the long axis of the tube which is placed along *Y*-axis in Figure 2(a)) of resolution phantom where the long axis of the tubes is orthogonal to the acquisition plane and LR image stacks were collected with a voxel AR of 1 : 1 : 10: (a) interpolated, (b) shifted, (c) rotational, (d) orthogonal, (e) inplane, and (f) line plot, and where the long axis of the tubes is oblique to the acquisition plane: (g) interpolated, (h) shifted, (i) rotational, (j) orthogonal, (k) inplane, and (l) line plot.

Short-axis images of the resolution phantom (voxel AR = 1 : 1 : 5) where the long axis of the tubes were positioned along the *Y*-axis of Figure 2 are shown in Figures 5(a)–5(e). The lack of resolution in the slice-select direction is apparent in Figure 5(a), where the 2D images are acquired at a slice thickness greater than the distance between the tubes, and linear interpolation is used for reconstruction. Figures 5(b)–5(d) are the corresponding short axis images from the SRR images based on shifted, rotated, and orthogonal acquisition geometries, respectively. The five tubes are resolved in the SRR images based on all three acquisition geometries, however a significant blurring is observed in the slice-select direction for the SRR image based on parallel shifts (Figure 5(b)) and to a lesser extent for the SRR image based on rotational acquisition (Figure 5(c)). The SRR image based on orthogonal acquisition (Figure 5(d)) reproduced the five tubes with the least amount of blurring artifact and looked similar to that observed in the inplane short-axis image (Figure 5(e)), where the sampling rate is great enough to resolve the tubes in the image. The intensity line plot shown in Figure 5(f) illustrates a decrease in peak intensities in the SRR images relative to that observed for the inplane image, with the least amount of change observed in the SRR image based on the orthogonal acquisition geometry. Similar results were observed for the SRR HR images when the LR image stacks were collected with a voxel AR of 1 : 1 : 10 (Figure 6).

Short axis images of the line pair phantom (voxel AR = 1 : 1 : 5) where the long axes of the tubes were aligned oblique to the slice-select direction of the acquisition geometries are shown in Figures 5(g)–5(k). The lack of resolution in the slice-select direction is observed in Figure 5(g), where the 2D images are acquired at a slice thickness greater than the distance between the spaced tubes. The five tubes are not resolved in the reconstruction based on the parallel shift acquisition geometry (Figure 5(h)) but are resolved in the reconstructions based on rotational (Figure 5(i)) and orthogonal (Figure 5(j)) acquisition geometry. However, blurring is observed in the slice-select direction of the SRR image based on rotational acquisition geometry but not in the SRR image based on orthogonal acquisition geometry. This is better illustrated in the intensity line plot presented in Figure 5(l). Similar results were observed for low resolution data sets collected with a voxel AR of 1 : 1 : 10 (Figure 6).

### 3.2. Biological Phantom

**Figure 7:**
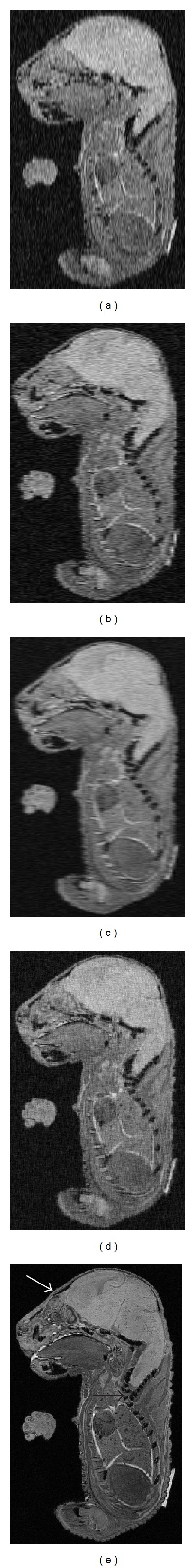
2D sagittal view of SRR images of the *ex vivo *embryo based on different acquisition geometries: (a) interpolated, (b) shifted, (c) rotational, (d) orthogonal, and (e) isotropic. White arrow indicates structures in the nasal cavity not clearly observed in the corresponding SRR images.

Increased resolution of biological structures in the *ex vivo* embryo was observed in the SRR images over a single LR image stack using straight linear interpolation (Figure 7). This was true for the SRR images based on LR image stacks acquired at both 0.19 mm (AR = 1 : 1 : 4) and 0.26 mm (AR = 1 : 1 : 6) slice thicknesses. Small structures (1-2 mm in width), such as those highlighted in the sinuses and the vertebrae, were not as clearly delineated in the SRR images as those observed in the isotropically acquired 3D image (Figure 7(e)).

The SRR image based on rotational geometry appeared more smooth than those based on the shifted and orthogonal geometries. This smoothing effect increased when the slice thickness of the LR images was increased from 0.19 (AR of 1 : 1 : 4) to 0.26 mm (AR of 1 : 1 : 6). Streaking artifacts were observed in uniform regions of the SRR images based on shifted (Figure 7(b)) and orthogonal (Figure 7(d)) geometries but were not as apparent in the SRR image based on rotational geometry (Figure 7(c)). These streaking artifacts were observed in the direction of linear interpolation used for upsampling in the LR direction. 

**Figure 8:**
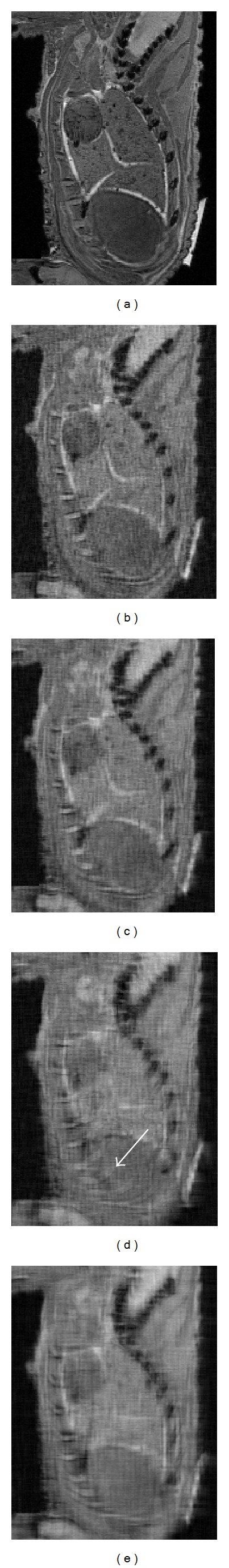
2D sagittal view of the *ex vivo *embryo for (a) 3D isotropic acquisition and SRR images based on LR image stacks with AR equal to (b) 1 : 4, (c) 1 : 6, (d) 1 : 8, and (e) 1 : 10. White arrow highlights rib structures that are present in the SRR image but not present in the isotropic 3D image.

The SRR images based on orthogonal acquisition for different voxel ARs are shown in Figure 8. The SRR images exhibited increased streaking artifacts with increasing slice thickness. Once the slice thickness was increased beyond a voxel AR of 1 : 1 : 6, we observed structures from adjacent slices that were not located in their proper through-plane location (Figure 8(d)). This artifact was not consistently observed with increasing slice thickness, as can be seen in Figure 8(e), suggesting the artifact is dependent upon where those structures are positioned in the original LR sampling. 

SNR, CNR, mean edge width, and acquisition time for the SRR images and the isotropically acquired image of the *ex vivo* embryo are listed in Table 1. SNR and CNR increased for SRR images with increasing voxel AR. The SNR and the CNR for the SRR images were greater for the SRR images based on the orthogonal geometry followed by SRR images based on the rotated and shifted geometries. Mean edge width was similar for SRR images with voxel ARs of 1 : 1 : 4 and 1 : 1 : 6, but an increase was observed for the SR images based on orthogonal geometry at the increased voxel ARs of 1 : 1 : 8 and 1 : 1: 10. 

### 3.3. *In Vivo* Mouse

**Figure 9:**
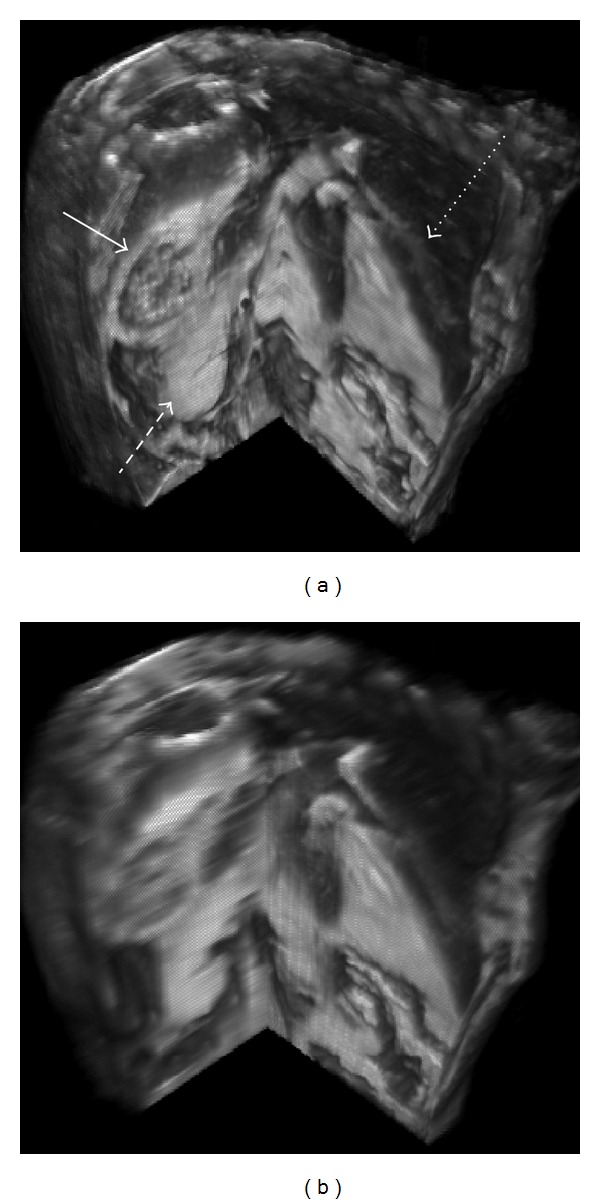
Cutaway section from 3D volume rendering of the *in vivo *mouse abdomen based on orthogonal SRR (a) with AR equal to 1 : 1 : 10 and single interpolated view (b). The solid arrow points to the wall of the stomach, dashed arrow to the kidney, and dotted arrow to the liver vasculature. Biological structures can be observed clearly in any oblique cutting plane of the SRR image as opposed to single 2D multislice image with linear interpolation.

A 3D volume rendering of the SRR image of the live mouse is presented in Figure 9. Biological structures, such as the wall of the stomach, kidneys, and liver vasculature are clearly observed in all three image planes of the SRR image. A 3D volume rendering based on the sagitally acquired LR image with linear interpolation illustrates the loss of image quality in planes other than the primary HR acquisition plane. The streaking artifacts normally observed in the 2D slice view of the SRR images obtained from orthogonal acquisition are not observed in the volume rendered images. The total time to acquire all three LR image stacks used for the *in vivo* SRR was 45 minutes due to the respiratory and cardiac gating. A full 3D isotropic scan of this mouse would have taken more than 4 hrs with gating and would not be possible for live animal applications.

## 4. Discussion

The results from this study illustrate that SRR using multiple LR views improves image content and spatial resolution in the slice-select direction of 2D multislice acquisitions. Increased SNR and CNR were observed in the SRR images from the orthogonal acquisition compared to those reconstructed using shifted and rotational geometries. SRR images based on rotational acquisition geometry exhibited a smoothing of the edges in both the resolution and biological phantom. This was observed visually and in the mean edge width calculated from the SRR images. However, streaking artifacts were observed in the SRR images based on shifted and orthogonal geometries that became more pronounced at the higher ARs of 1 : 1 : 8 and 1 : 1: 10. These streaking artifacts appear to be due to the linear interpolation used for upsampling the LR images and updated differences in LR and predicted HR images. The use of higher order or standard sigmoid-shaped interpolation kernels did not improve this streaking artifact. 

Streaking artifacts may not be as apparent in the SRR images based on rotational geometry because the linear interpolation is occurring at oblique angles to the view plane or they may be averaged “out” due to the number of rotational angles used for the SRR. Streaking artifacts were only observed in 2D slice views of the SRR images and not in the volume rendered images. This suggests that the ray tracing used for creating the volume rendered image is also averaging “out” the appearance of the streak artifacts. 

 The main advantage of using orthogonal acquisition for SRR over the other proposed acquisition geometries is that it requires the minimum number of views and thus the minimum amount of acquisition time. Additionally, orthogonal or nearly orthogonal acquisitions are typically acquired in most clinical and small animal imaging applications. SRR based on orthogonal views may result in better 3D volumes than those based on the other two geometries because the high resolution volume space is more uniformly sampled in all three directions.

Theoretically, SRR images based on three views is an underdetermined problem when the slice thickness is three times greater than the inplane voxel size (AR = 1 : 1 : 3). Practically, the image quality of SRR images based on the orthogonal geometry and limited number of views was not significantly affected until the slice thickness of the LR image stacks was greater than six times the inplane voxel size (AR = 1 : 1 : 6). This was also observed in orthogonal super resolution reconstructions of a digital brain phantom by Gholipour et al. [9]. 

Whole body mouse phenotyping is typically performed in *ex vivo* specimens [1]. However, phenotyping in live animals has significant advantages in that you can observe structures in their native environment and monitor changes in structure and function over time. The main factors that affect the acquisition of HR images in live mice are the large field-of-view required for the whole body and the gated acquisitions required for respiratory and cardiac motion. We have successfully demonstrated that SRR can be implemented in a live animal model that requires respiratory- and ECG-gating to account for motion. A full 3D isotropic acquisition of the mouse used in this study would have taken more than 4 hrs with gating and would not be possible in a live animal imaging setting. This SRR acquisition was limited to an AR of 1 : 1 : 10 which is common for 2D multislice *in vivo* imaging applications. Visual comparison of different phenotypes using volume rendering would be possible at this resolution however image postprocessing such as object segmentation and quantitative analysis may suffer from the reconstruction artifacts observed in SRR images obtained at higher ARs. 

SRR has been shown to be useful in clinical applications where images are corrupted by motion such as fetal brain imaging *in-utero* [10–12] and imaging of the tongue [13]. These approaches use registration to align the data to an anatomical model. Gholipour et al. [10] developed a model based super resolution reconstruction framework based on arbitrarily oriented slices in 3D acquisition space. This algorithm was applied to volume reconstructions from fetal brain MR images where interslice motion is prevalent. Rigid body registration was used to correct interslice motion using a slice-to-volume registration approach. Although this approach has shown to be effective using 2D acquisitions from arbitrary orientations, they have also suggested using multiple orthogonal or overlapped slice acquisitions for high resolution reconstructions. Woo et al. [13] used an orthogonal SRR approach to obtain high resolution 3D images of the tongue. Super resolution offered a viable alternative to obtain 3D volumes where acquisition time is limited by the involuntary motion of the tongue. 

SRR has also recently been implemented for improving spatial resolution in DW imaging of the human brain using single-shot EPI acquisition protocols [14]. Spatial resolution in DW imaging is inherently low relative to the structures of interest and isotropic acquisition at high spatial resolution is virtually impossible due to the long scan times required for data acquisition. Although improvements to hardware and acquisition protocols have been implemented to address this problem, it still remains a challenge to obtain high resolution isotropic DW images. 

In this work IBP was used for reconstructing the SRR images. More recently, regularized least square methods that incorporate prior knowledge as a regularization term have been proposed [15] for SRR implementation. These different optimization algorithms, such as LASR and Tikhonov regularization (TIK), have shown improved resolution over IBP optimization when the number of LR stacks used for reconstruction is greater than three (i.e., TIK) [8]. However, SNR was observed to be greater for IBP when a larger number of LR stacks was used for reconstruction. The results of Plenge's study suggest that different optimization schemes may perform better than others and may be dependent upon the application and the number of LR stacks used for reconstruction. Therefore, future work will focus on implementing these optimization schemes and testing them in our phenotyping models as well as developing techniques for reducing the streaking artifacts observed in the SRR images based on orthogonal acquisition geometry. These techniques should also help to reduce misregistration of structures observed in SRR images from higher ARs. 

## 5. Conclusion

We have shown that the SRR images based on orthogonal acquisition geometry provide a better tradeoff between resolution, acquisition time, SNR, and CNR than those based on shifted and rotational acquisition geometries. This was observed for LR images with voxel ARs less than 1 : 1 : 6. However, for the orthogonal acquisition geometry, we observed when slice thickness was increased beyond a voxel AR of 1 : 1 : 6, artifacts resulted in the SRR image. As these artifacts were not consistently present in the same location with increasing voxel AR, we concluded that the artifact is dependent upon the sampling and where a specific slice occurs in the object being sampled. Finally, we demonstrated that SRR is applicable for *in vivo* gated acquisitions. This observation along with the possibility of applying the SRR algorithm with a higher voxel AR has the potential to make SRR a practical alternative for the acquisition of 3D HR isotropic images in small animal phenotyping applications.

## Figures and Tables

**Table 1:** Quantitative measures of image quality calculated from images of biological phantom.

AR	Int^b^	Shifted	Rotated	Orthogonal
SNR				
1 : 1 : 1^a^	26.8			

1 : 1 : 4	20.0	21.6	23.3	25.4
1 : 1 : 6	22.2	25.0	27.1	28.4
1 : 1 : 8				35.2
1 : 1 : 10				41.5

CNR				
1 : 1 : 1^a^	5.6			

1 : 1 : 4	5.1	5.3	6.0	6.9
1 : 1 : 6	5.8	6.8	7.2	8.0
1 : 1 : 8				7.9
1 : 1 : 10				7.9

Mean edge width (in HR pixels)				
1 : 1 : 1^a^	2.4			

1 : 1 : 4	5.9	4.2	3.7	3.2
1 : 1 : 6	6.1	4.1	3.8	3.5
1 : 1 : 8				3.9
1 : 1 : 10				4.4

Acquisition time (mins)				
1 : 1 : 1^a^	18.5			

1 : 1 : 4	3	12	18	9
1 : 1 : 6	3	18	18	9
1 : 1 : 8				9
1 : 1 : 10				9

^a^Isotropically acquired 3D image.

^b^Refers to linear interpolation from one LR image stack.
